# Evaluation of Fluidity and Strength of High-Early-Strength Cement-Based Repair Materials by Adding SB Latex and Wollastonite Mineral Fibers

**DOI:** 10.3390/ma16155239

**Published:** 2023-07-26

**Authors:** Yeon-Jae Choo, Jae-Hyuk Koo, Su-Jin Lee, Chan-Gi Park

**Affiliations:** 1 Department of Agricultural Engineering, Kongju National University, Yesan-eup 32439, Republic of Korea; chooyj621@contecheng.co.kr (Y.-J.C.); wogur7933@naver.com (J.-H.K.); 2 Department of Architectural Engineering, Keimyung University, Daegu 42601, Republic of Korea; sjlee@gw.kmu.ac.kr; 3 Department of Regional Construction Engineering, Kongju National University, Yesan-eup 32439, Republic of Korea

**Keywords:** fiber-reinforced cement-based materials, mechanical properties, repair materials, SB latex, wollastonite mineral fiber

## Abstract

Concrete structures often fail to perform their original functions due to problems such as deterioration and damage over time. Therefore, various repair materials have been studied to maintain deteriorated concrete structures. This study experimentally investigated the mechanical properties of high-early-strength cement-based repair materials for spraying. For spraying, the cement-based materials should have adoptable fluidity and strength: 200 ± 100 mm for flow; 20 MPa at 24 h and 40 MPa at 28 days for compressive strength, and 8 MPa at 28 days for flexural strength. Wollastonite mineral fibers (3–5 wt.%) and styrene–butadiene (SB) latex (5–7 wt.%) were studied to enhance this requirement. Fluidity was evaluated by flow test and measuring the heat of hydration; mechanical properties were evaluated in terms of compressive and flexural strength. The cement-to-silica sand ratio (C:S ratio) was also applied differently to adjust the pot life of polymer cement-based material (1:1 and 1:1.5) as a binder. Because wollastonite mineral fibers and SB latex affect workability, the water-to-binder ratio was regulated to reach the target flow according to the amount of wollastonite mineral fibers and SB latex. Regardless of the C:S ratio, all studied mixtures met the target 28 day compressive strength at 24 h, decreasing in strength with increasing amounts of wollastonite mineral fibers and latex. Flexural strength also fulfilled the target value, and it increased with increasing amounts of wollastonite mineral fibers and latex, unlike compressive strength. The optimal mix proportion of high-early-strength cement-based repair materials constituted 3 wt.% wollastonite mineral fibers and 5 wt.% SB latex as the binder in a C:S ratio of 1:1.5.

## 1. Introduction

Concrete structures are constructed to satisfy the required performance under various environmental conditions [1,2]. However, in numerous cases, they often fail to fulfill their original functions due to issues such as deterioration and damage over time [3]. The degradation of concrete can occur due to external environmental factors, such as carbonation, salt damage, chemical erosion, cracks, and freezing damage, and internal factors, such as alkali–aggregate reaction [4,5]. These factors combine to cause the deterioration of concrete such as cracks and peeling/exfoliation [6]. Because structural stability is eventually affected when exposed to continued deterioration, certain improved repair techniques are needed.

The lifetime of infrastructure can preferably be extended via repair, rather than by constructing new structures, provided that the safety of the structure can be ensured [7]. Accordingly, there is increasing interest in the development of repair materials and construction methods for deteriorated concrete structures. Various types of polymer-modified mortar are generally applied for concrete repair [1]. Such repair materials can provide excellent mechanical properties and durability [8]. Notably, polymer-modified mortar can be applied by spraying, which is a commonly used method for repair. However, there is a strong likelihood of cracking when the spray method is used, and the crack can increase permeability and lead to a decrease in durability [9]. Therefore, the crack control of polymer-based repair material needs to be enhanced.

Ultrarapid hardening cement was evaluated in a study to obtain high early strength, in spite of a higher heat of hydration than Type I cement. The higher heat of hydration not only develops strength rapidly, but also leads to cracks [10]. Thus, wollastonite mineral fiber was used as reinforcing fiber to control cracks, and styrene–butadiene (SB) latex was used to provide sufficient fluidity for enhancing clogging by fiber balls [11]. It is known that reinforcing fibers improve the resistance to cracking of cement-based materials; thus, fiber-reinforced cement-based materials can suppress the occurrence and growth of cracks through fiber debonding, failure, and bridging effects [12,13,14]. However, fiber balling reduces not only the workability but also the strength of concrete. On the other hand, wollastonite mineral fibers can solve this problem because it has a smaller aspect ratio than synthetic fibers and steel fibers used in existing fiber-reinforced concrete [15,16,17,18].

The SB latex polymer is expected to improve watertightness, carbonation, and resistance to freezing and thawing by improving the pore structure of the cement repair material [11]. Furthermore, it decreases the amount of rebound, thereby improving performance and adhesion to the existing structure without the thickener commonly used to enhance bonding for sprayed material. Through these advantages, it is possible to increase the construction speed and shorten the construction period compared to conventional materials.

This study aimed to evaluate the fluidity and mechanical properties of high-early-strength cement-based repair materials (HERM) by adding wollastonite mineral fibers and SB latex for the spray method. Furthermore, the effects of the amount of wollastonite mineral fibers and SB latex were also investigated.

## 2. Experimental Plan

### 2.1. Materials

**Figure 1:**
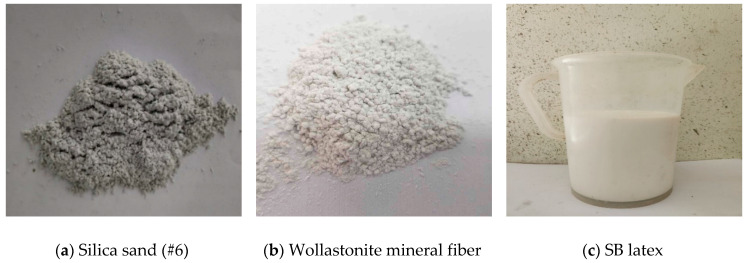
Studied materials.

Table 1 presents the chemical composition of the ultrarapid hardening cement used in this study. The physical properties of silica sand used as the fine aggregate are listed in Table 2. Wollastonite mineral fiber was used as reinforcement, with an aspect ratio ranging from 3 to 20, and its physical properties are shown in Table 3. The properties of the SB latex polymer are presented in Table 4. Figure 1 shows the studied materials.

### 2.2. Mix Proportions

The properties of the HERM were evaluated by considering mix ratios such as C:S ratios of 1:1 and 1:1.5, wollastonite mineral fiber contents of 0, 3, and 5 wt.% of the binder (cement + silica sand), and SB latex contents of 0, 5, and 7 wt.% of the binder. The C:S ratio used in the field (Dakyung Construction Co., Ltd., Guri, Gyeonggi, Republic of Korea) was applied, which was determined by investigating the mixing ratio of repair materials in Korea [19]. The SB latex and wollastonite mineral fiber were added to improve the performance of the repair material. Considering the setting time, the amount of retarder in all mix proportions was fixed at 1 wt.% of cement; the water/binder (W/B) ratio was adjusted (without adding plasticizer) to obtain the target flow of 200 ± 10 mm required for spraying. The designed compressive strength and flexural strength of the polymer mortar are 40 MPa and 8 MPa at 28 days according to KS F 2476 [20]. The studied mix proportions are listed in Table 5.

### 2.3. Experimental Methods

In order to suggest a cement-based repair material that satisfies the requirement for deteriorated concrete structures, using wollastonite mineral fibers and SB latex as additives, workability and mechanical tests were conducted to experimentally determine the optimal amount of SB latex and wollastonite mineral fiber.

#### 2.3.1. Flow Test

**Figure 2:**
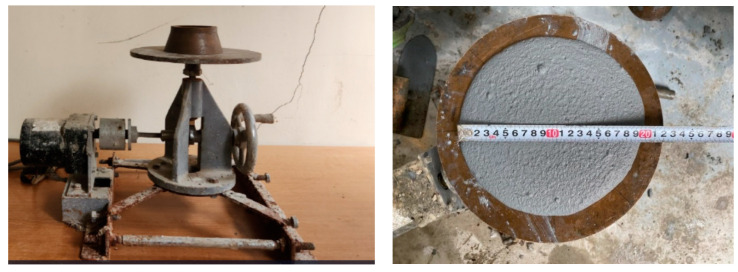
Flow value measurement.

The flow test was carried out according to KS F 2476 [20] to confirm the workability of the repair material. Briefly, the flow cone was placed upright in the center of the flow table and filled with two layers of mortar. Each layer was tamped 15 times over the entire surface; the front tip of the tamping rod penetrated approximately half the depth of the lower layer, while the upper layer was leveled with mortar [20]. The flow cone was immediately lifted up, and then the flow table moved in a falling motion 15 times over an interval of 15 s [20]; the diameter of the spread was measured. The target flow value was 200 ± 10 mm to ensure suitability for the spraying equipment. Figure 2 shows the flow value measurement.

#### 2.3.2. The Heat of Hydration Measurement

**Figure 3:**
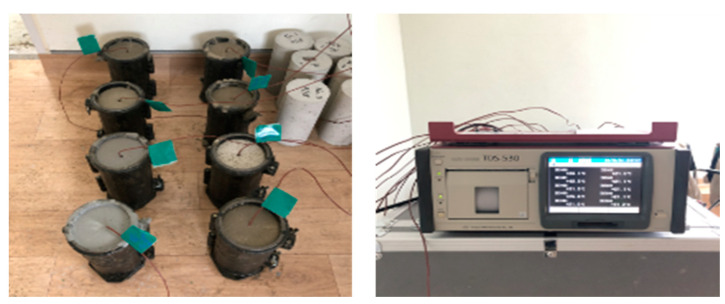
The heat of hydration measurement.

The hydration of cement is an exothermic process. Cement-based materials have low thermal conductivity and, consequently, delay the heat of hydration dissipation to the external environment. Notably, ultrarapid hardening cement hydrates with a higher temperature of the heat of hydration develop early strength; however, the higher initial internal temperature can lead to cracking [21,22]. The initial crack occurs in a local part subjected to a big difference in temperature [21,22]. By measuring the heat of hydration, the degradation of the strength of the repair materials, as well as the change in the internal temperature of the cement-based materials, was confirmed and analyzed. A thermocouple was embedded in the center of the cylindrical specimen sized Ø100 × 200 mm, and the temperature was measured every 5 min for 24 h. Figure 3 shows the heat of the hydration measurement setup.

#### 2.3.3. Compressive Strength

**Figure 4:**
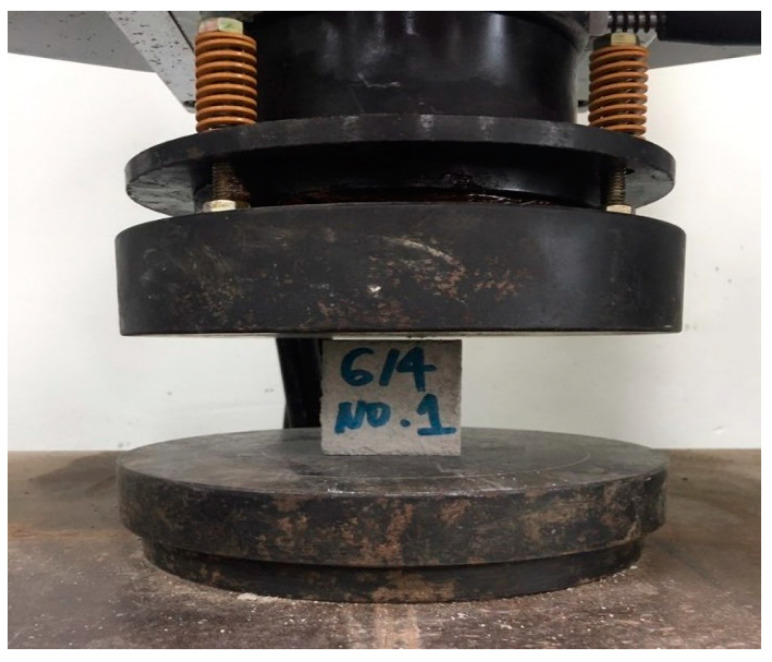
The compressive strength test setup.

The compressive strength of the repair material was determined by KS L 5105 [23]; the target values were 20 MPa at 1 day and 40 MPa at 28 days, which correspond to the repair material currently commercially available in Korea. A cubic specimen sized 50 mm × 50 mm × 50 mm was prepared and initially cured for 24 h. After initial curing, the demolded specimen was cured underwater, and compressive strength was measured at 1, 7, and 28 days (Figure 4). The compressive strength was measured twice for each age of the material for accuracy, and the mean strength under each condition was reported.

#### 2.3.4. Flexural Strength

**Figure 5:**
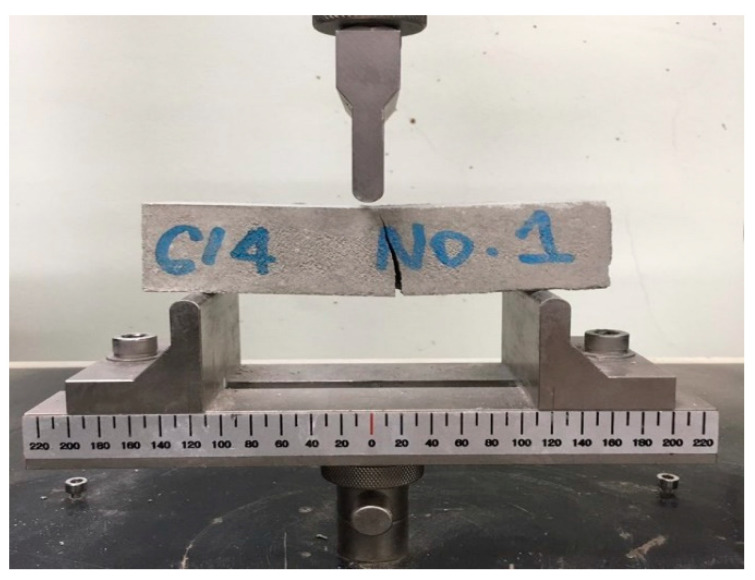
The flexural strength test setup.

The flexural strength of the repair material was determined in accordance with KS F 2476 [20]. Three prismatic specimens sized 40 mm × 40 mm × 160 mm were prepared for each mixture. The specimens were cured under the same conditions as the compressive strength specimens. The flexural strength was measured at 7 and 28 days, with a target strength of 8 MPa at 28 days (Figure 5). It was measured twice for each age of the material for accuracy, and the mean strength under each condition was reported.

## 3. Experimental Results

### 3.1. Flow Test

**Figure 6:**
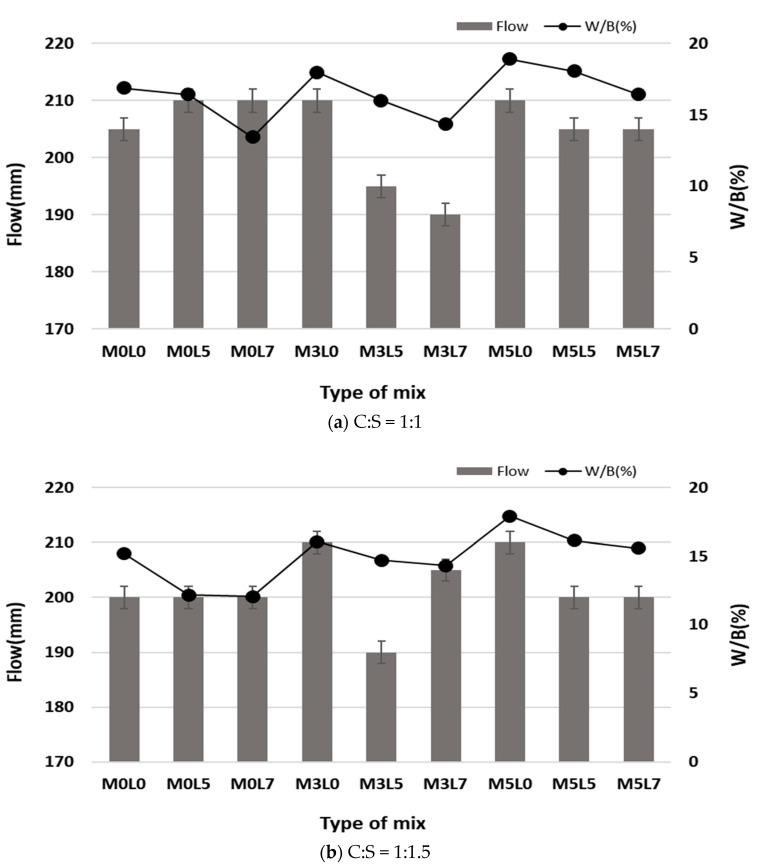
Flow according to cement and silica sand ratio.

Figure 6 shows the W/B ratio when the target flow value of 200 ± 10 mm was satisfied (Figure 6a for C:S = 1:1, Figure 6b for 1:1.5). The flow patterns were similar with respect to wollastonite mineral fiber and SB latex levels. The W/B ratio required to satisfy the target flow value increased with increasing amounts of wollastonite mineral fiber, whereas there was no change in flow value upon increasing the amount of latex. When wollastonite mineral fiber and latex were added together, the W/B ratio required to satisfy the target flow value decreased with the usage of latex increasing from 5% to 7% for mixtures with wollastonite mineral fiber amounts of 3% and 5%.

For similar workability, the W/B ratio increased with increasing amounts of wollastonite and decreased with increasing amounts of latex. The surfactant and polymer particles of the latex improved workability. However, the needle-shaped fine wollastonite fibers increased interactions within the cement paste; thus, similar workability was achieved by increasing the W/B ratio as the amounts of wollastonite fibers increased [24,25]. The W/B ratio for the C:S ratio of 1:1.5 was always lower than that for the 1:1 case. This means that the rough surface of silica sand particles was partially accounted for [26]. The unit weight of cement for a C:S = 1:1.5 mix was lower than that for a 1:1 mix; thus, it was smaller than the required unit quantity to secure the target flow value of a mixture at C:S = 1:1.5 than for one at 1:1.

### 3.2. The Heat of Hydration Measurement

**Figure 7:**
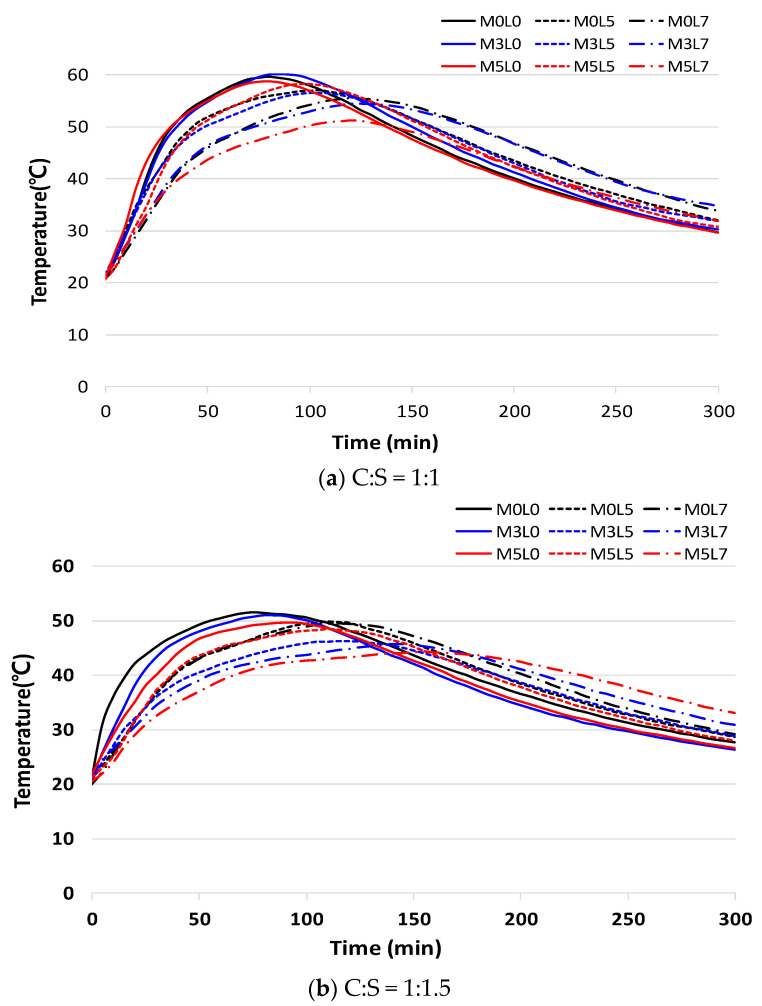
The heat of hydration according to the C:S ratio.

**Figure 8:**
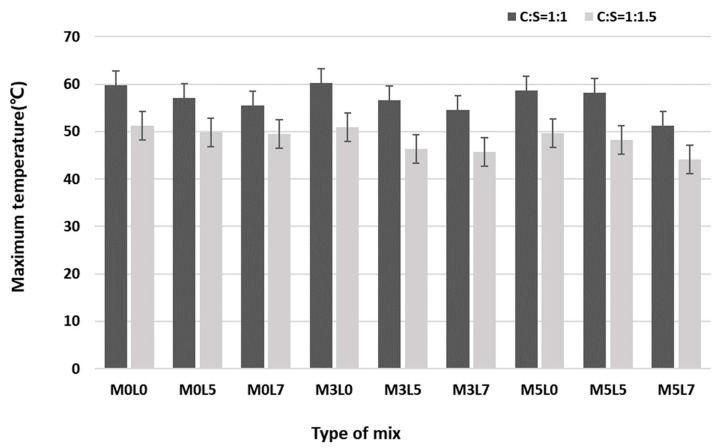
Maximum temperature of hydration according to the mix proportions.

Figure 7 shows the time–temperature curves for all studied mix proportions (Figure 7a for C:S = 1:1; Figure 7b for 1:1.5). According to measurements of the heat of hydration for 24 h, the curves show that all mixes reached maximum temperature within 3 h, and the heat of hydration continued to decline at 5 h. Similar trends were observed at the two C:S ratios. Increasing the amounts of wollastonite fibers only slightly decreased or had no effect on the heat of hydration, compared with the control without wollastonite fibers and latex (M0L0). The addition of more latex significantly decreased the heat of hydration. For the mixtures containing 3 and 5 wt.% wollastonite fibers, increasing the amount of latex from 0 to 5 or 7 wt.% decreased the heat of hydration. The heat of hydration was, thus, marginally affected by the addition of wollastonite fibers only; it was strongly affected by amounts of latex, regardless of whether latex was combined with wollastonite fibers. These results confirm that latex delayed the hydration reaction. Previous studies have noted that wollastonite mineral fiber decreases the heat of hydration [27]. Figure 8 shows a comparison of the maximum temperature within the mortar for the various mixes. The maximum temperature for the C:S of 1:1.5 mixtures was 15% lower than that for the 1:1 mixtures because the unit weight of cement was less than C:S of 1:1.

#### 3.2.1. Compressive Strength

**Figure 9:**
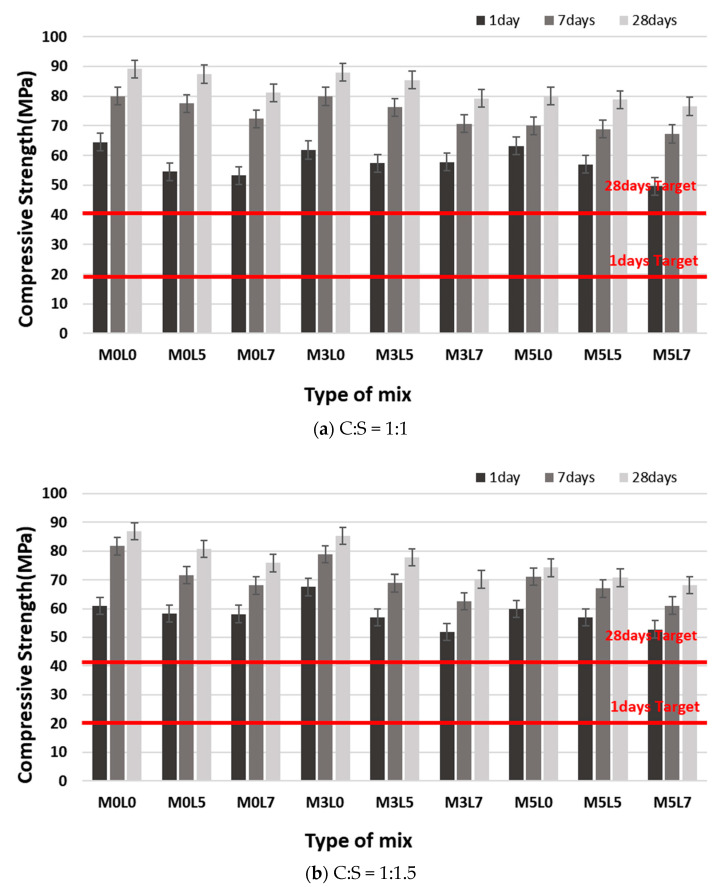
Compressive strength with age.

**Figure 10:**
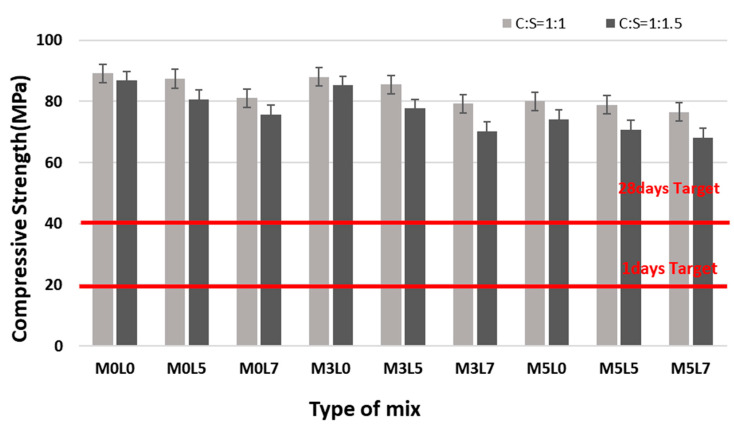
Comparison of 28 day compressive strength according to the C:S ratio.

Figure 9 shows the change in compressive strength with age. The C:S 1:1 and 1:1.5 mixes were similarly affected by the addition of wollastonite and latex (Figure 9a for 1:1; Figure 9b for 1:1). All mixes satisfied the target compressive strength of 20 MPa at 1 day and 40 MPa at 28 days. The compressive strength decreased with increasing amounts of wollastonite fibers and latex because the unit weight of cement decreased with increasing amounts of wollastonite fibers and latex. This resulted in weaker bonding between the polymer and the aggregate, leading to lower compressive strength [16,28]. Suppression of the hydration reaction by a latex film also decreases compressive strength [16,28]. Figure 10 shows a comparison of the compressive strength at 28 days for the various mix proportions. Mixes at the 1:1 and 1:1.5 ratios satisfied the target strength at 28 days. However, the compressive strengths of the mixes at 1:1.5 were approximately 10% lower than those of the 1:1 mixtures due to the use of a different unit weight of cement.

#### 3.2.2. Flexural Strength

**Figure 11:**
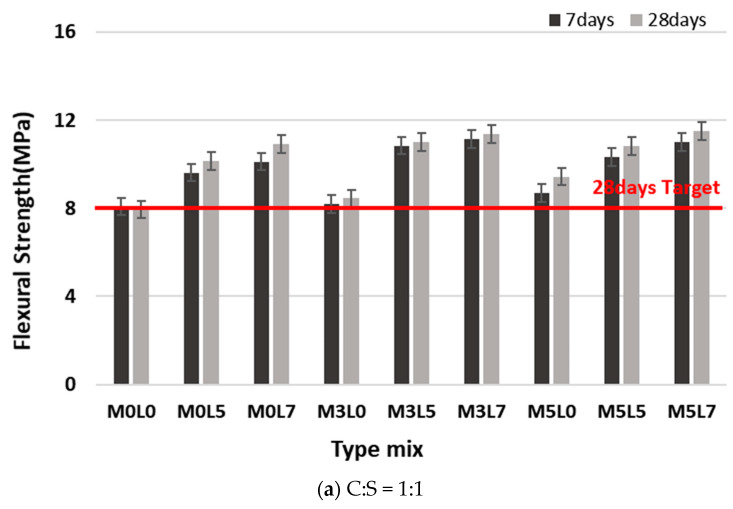

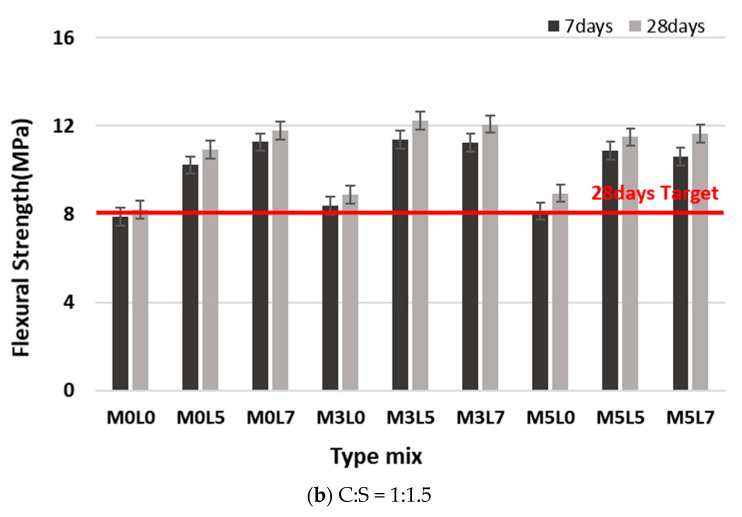
Comparison of flexural strength with age.

**Figure 12:**
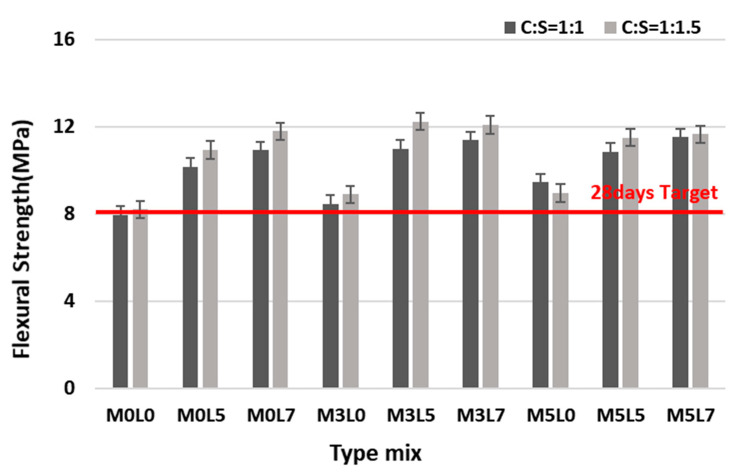
Comparison of 28 day flexural strength according to the C:S ratio.

Figure 11 compares the flexural strength of the various mixes. All mixes satisfied the target flexural strength of 8 MPa at 28 days. Mixes at the C:S ratios of 1:1 and 1:1.5 were similarly affected by wollastonite fibers and latex. Flexural strength increased with the increasing addition of wollastonite fibers and latex. When amounts of wollastonite fibers were fixed at 3 and 5 wt.% and the amount of latex was increased from 0 to 5 or 7 wt.%, the flexural strength increased with increasing latex addition from 0 to 5 wt.%. However, it was not significantly higher than for the mix with 7 wt.% latex. For the C:S ratio of 1:1.5, the flexural strength of the M3L5 and M3L7 mix was higher than that of the M5L5 and M5L7 mix. Figure 12 compares the flexural strength at 28 days for the various mixes. All mixes at the 1:1 and 1:1.5 ratios satisfied the target flexural strength at 28 days.

#### 3.2.3. Determination of Mixing Ratio

As a result, Table 6 suggests the optimal mix proportion as a repair material satisfying the target properties for the spray method: 200 ± 10 mm for flow, 20 MPa at 1 day and 45 MPa at 28 days for compressive strength, and 8 MPa at 28 days for flexural strength.

## 4. Conclusions

This study evaluated the properties of high-early-strength cement-based repair materials containing wollastonite mineral fiber and SB latex when the binder ratio differed through flow, along with the heat of hydration; compressive and flexural strength tests were also conducted. This study suggested a mixed proportion of the repair materials. Some conclusions are drawn below.

As the flow test result, the W/B ratio required to satisfy the target flow value increased with the increasing amount of wollastonite fibers, but decreased with the increasing amount of SB latex at C:S ratios of 1:1 and 1:1.5. When only wollastonite mineral fibers were added, a higher W/B ratio was needed to satisfy the target flow value, while the amounts of SB latex could be reduced such that the target flow value was attained.

The heat of hydration measurements for C:S ratio mixes of 1:1 and 1:1.5 confirmed that the heat of hydration did not significantly decrease compared with the M0L0 (the control) when increasing the addition of wollastonite fibers. However, mixes that added SB latex only or wollastonite fibers and SB latex together showed a lower temperature of the heat of hydration, and the temperature curves rose gently. Notably, this pattern was clear for the C:S = 1:1.5 mixes.

All studied mixtures met the target compressive strength of at least 45 MPa at C:S ratios of 1:1 and 1:1.5. The compressive strength decreased with increasing amounts of wollastonite fibers and SB latex. The compressive strength of C:S = 1:1 mixes was higher than that of C:S = 1:1.5 mixes.

For flexural strength, all mixtures met the target of at least 8 MPa for C:S 1:1 and 1:1.5 mixes. Notably, the flexural strength increased with increasing amounts of wollastonite mineral fibers and SB latex. In the case of the C:S = 1:1.5 mix, the flexural strength of the M3L5 and M3L7 mixes was higher than that of the M5L5 and M5L7 mixes. The flexural strength of the C:S = 1:1.5 mixes was 5% higher than that of the C:S 1:1 mix.

The determined mix proportion satisfied the target properties of high-early-strength cement-based repair materials using 3 wt.% wollastonite fiber and 5 wt.% SB latex for a C:S ratio of 1:1.5.

## Figures and Tables

**Table 1:** Chemical composition of ultrarapid hardening cement (Gmaxrefid Co., Ltd., Namyangju, Gyeonggido, Republic of Korea).

Chemical Composition (%)							Blaine Fineness (cm^2^/g)	Specific Gravity
SiO_2_	Al_2_O_3_	Fe_2_O_3_	CaO	MgO	K_2_O	SO_3_		
13 ± 3	17.5 ± 3	3>	50 ± 3	2.5>	0.21	14 ± 3	5400	2.95

**Table 2:** Physical properties of silica sand (Samwon Chemical Co., Ltd., Seoul, Republic of Korea).

No.	Size (mm)	Density (g/mm^3^) (20 °C)	Organic Impurities	Finess Modulus
6	≤0.3	2.62	Nil	1.95

**Table 3:** Properties of wollastonite mineral fiber (Samwon Chemical Co., Ltd., Seoul, Republic of Korea).

Properties	Values
Appearance	White
Shape	Acicular
Length (mm)	0.4–0.6
Transverse dimension (µm)	25–150
Range of aspect ratio	3–20
Coefficient of expansion (mm/mm/°C)	6.5 × 10^−6^
Density (g/mm^3^)	2.9
Water solubility (g/100 cc)	0.0095
pH	9.9

**Table 4:** Properties of SB latex (Joongang polytech Co., Ltd., Yangsan, Gyeongnam Republic of Korea).

Solids Content (%)	Styrene Content (%)	Butadiene Content (%)	pH	Density (g/mm^3^)	Surface Tension (dyne/cm)	Particle Size (Å)	Viscosity (cps)
49	34 ± 1.5	66 ± 1.5	11.0	1.02	30.57	1700	42

**Table 5:** Mix proportions.

C:S Ratio	Type of Mix	W/B (%)	Used Weight (kg/5 L)					Used Weight (g/5 L)		Flow (mm)
			Water	Binder (B)		Wollastonite Fiber	SB Latex	Plasticizer	Retarder	
				Cement	Silica Sand					
1:1	M0L0	16.87	1.63	4.84	4.84	0.00	0.00	0.00	96.8	205
	M0L5	16.43	1.59				0.48	14.52		210
	M0L7	13.43	1.30				0.68	20.33		210
	M3L0	17.98	1.74			0.29	0.00	0.00		210
	M3L5	16.01	1.55				0.48	14.52		195
	M3L7	14.36	1.39				0.68	20.33		190
	M5L0	18.90	1.83			0.48	0.00	0.00		210
	M5L5	18.08	1.75				0.48	14.52		205
	M5L7	16.43	1.59				0.68	20.33		205
1:1.5	M0L0	15.2	1.56	4.11	6.16	0.00	0.00	0.00	102.63	205
	M0L5	12.2	1.25				0.51	15.39		210
	M0L7	12.1	1.24				0.72	21.55		210
	M3L0	16.1	1.65			0.31	0.00	0.00		210
	M3L5	14.7	1.51				0.51	15.39		195
	M3L7	14.3	1.47				0.72	21.55		190
	M5L0	17.9	1.84			0.51	0.00	0.00		210
	M5L5	16.2	1.66				0.51	15.39		205
	M5L7	15.6	1.60				0.72	21.55		205
	M5L0	17.9	1.84			0.51	0.00	0.00		210
	M5L5	16.2	1.66				0.51	15.39		205
	M5L7	15.6	1.60				0.72	21.55		205

**Table 6:** Determination of mix ratio.

Type of Mix	W/B (%)	Unit Weight (kg/m^3^)			Wollastonite Mineral Fibers	SB Latex
		Water	Binder (B, C:S = 1:1.5)			
			Cement	Silica Sand (#6)		
M3L5	12.2	222	728	1092	B × 3 wt.%	B × 5 wt.%

## Data Availability

Not applicable.
